# A new look at arthritis management: nociceptive reflex excitability as a pain biomarker

**DOI:** 10.1186/s13075-015-0821-0

**Published:** 2015-10-28

**Authors:** Carol A. Courtney

**Affiliations:** Department of Physical Therapy, University of Illinois at Chicago, 1919 W Taylor St, 4th Floor, Chicago, IL 60612 USA

## Abstract

Research studies have begun to identify altered neurophysiological mechanisms that develop as a consequence of chronic painful knee arthritis. Neuroplastic changes may occur in nociceptive pathways, thereby contributing to heightened chronic pain and spread of symptoms, as well as diminished quality of life. One biomarker for examining central sensitization of nociceptive pathways is the measurement of flexion reflex excitability, also called the nociceptive reflex. In a recent article in *Arthritis Research & Therapy*, Rice and colleagues utilize this biomarker to analyze the clinical and neurophysiological effects of a very common and typical medical intervention, joint aspiration and intra-articular corticosteroid injection.

## Editorial

Our understanding of the mechanisms underlying osteoarthritis (OA) has expanded greatly in recent years. In the past, much of the focus had been directed at cartilaginous changes occurring at the joint. However, researchers have suggested that OA may be better described as a ‘disease of the knee joint organ’, a process that affects not only cartilage, but also bone, ligament, synovium and periarticular musculature. This seems appropriate considering that muscle weakness, sensory changes, altered motor control and functional deficits are characteristic of this chronic condition. Not surprisingly, subsequent studies have begun to explore the neurophysiological changes that occur as a result of chronic painful knee arthritis. They have discovered that, over time, neuroplastic changes may occur in nociceptive pathways, thus contributing to accentuated chronic pain. One biomarker for examining central sensitization of nociceptive pathways is the measurement of flexion reflex excitability, otherwise known as the flexor withdrawal reflex. With chronic OA, this pain reflex may be more easily elicited. Therefore, it may serve as a biomarker indicating an increased propensity for pain flare-ups in this population. As a result, various clinical findings may be more readily found, such as expansion of pain distribution, heightened pain, and facilitated flare response. In a recent article in *Arthritis Research & Therapy,* Rice and colleagues [1] utilize this biomarker to analyze the clinical and neurophysiological effects of a very common and typical medical intervention, joint aspiration and intra-articular corticosteroid injection.

Intra-articular corticosteroid injection is a non-surgical medical intervention for painful knee arthritis that came into practice in the 1950s [2] for the purpose of decreasing inflammation and symptom relief. While not typically considered an inflammatory disease, OA is characterized by intermittent but recurrent inflammatory flares. Synovial inflammation is believed be the source of patient symptoms such as pain and stiffness. In fact, recent studies utilizing non-contrast magnetic resonance imaging (MRI) found a moderate relationship between reduction in synovitis and relief of pain [3, 4], while a study employing contrast MRI, which allows for better discrimination of synovitis, found a strong relationship between synovial tissue volume and pain [5]. Thus, synovitis is considered a treatment target in the medical management of arthritis. The study by Rice and colleagues [1] provides valuable insight on knee arthritis pain by drawing attention to the potential mechanisms of facilitation/depression of nociceptive excitability, and their clinical correlates, such as pain and altered muscle function. They found that the flexion reflex threshold increased significantly (indicating diminished excitability) immediately after the aspiration of fluid from the joint with a larger increase 5 and 15 days after subsequent corticosteroid injection. Experimental joint effusion rarely evokes sensations of pain in humans, indicating that effusion alone is unlikely to be the source of patient symptoms [1]. Accordingly, they concluded that in the presence of synovitis, aspiration likely reduces the discharge of knee joint afferents, all of which have the potential to influence flexion reflex excitability.

Rice et al. [1] found a further increase in the flexion reflex threshold 5 to 15 days following intra-articular corticosteroid injection. In addition to postulating on the ability of corticosteroid injection to dampen the nociceptive barrage to the central nervous system, the authors also proposed a novel hypothesis that corticosteroid injection might augment descending pain inhibition. Descending pain inhibition, measured via conditioned pain modulation, has been found to be impaired in the knee OA population [6], and this impairment may occur because of an imbalance in supraspinal mechanisms which are both facilitatory and inhibitory. Thus, removal of nociceptive stimuli may ‘normalize’ this asymmetry by lessening pain facilitation. However, the converse may be just as important in our management of OA pain. Exercise has been demonstrated to produce widespread hypoalgesia effects, perhaps through enhancement of descending pain inhibition. Exercise-induced hypoalgesia and conditioned pain modulation share similar characteristics and may share neurophysiologic mechanisms [7]. Therefore, the promotion of exercise, for the purpose of enhancing pain inhibition, is paramount in this population for control of chronic pain, in addition to its many other benefits. Finally, other therapeutic interventions, such as transcutaneous electrical nerve stimulation [8], manual therapy and dry needling/acupuncture, should be considered for their potential impact on descending pain modulation. This is particularly important as one reflects on the potential side effects of corticosteroid injections.

One of the more interesting findings of this study is the effect of joint aspiration and corticosteroid injection on measures of quadriceps strength. In this study, the authors demonstrated significant increases in quadriceps torque immediately following joint aspiration as well as at all subsequent measurement time points. Quadriceps weakness is a common complaint in individuals with knee arthritis, resulting in diminished function and disability [9]. The cause of this weakness has been debated and is likely multi-factorial in nature. In fact, various researchers have suggested quadriceps weakness to be both a cause and a consequence of OA. The findings of Rice et al. indicate that the underlying mechanism, at least in part, may be due to aberrant nociceptive processing, which is likely to occur at both spinal and supraspinal levels [10]. Further investigation examining the causes of OA-related quadriceps weakness would be valuable as remediation of this impairment is a prime objective in most rehabilitation protocols.

## Abbreviations

MRI: Magnetic resonance imaging
OA: Osteoarthritis
